# SNPAnalyzer 2.0: A web-based integrated workbench for linkage disequilibrium analysis and association analysis

**DOI:** 10.1186/1471-2105-9-290

**Published:** 2008-06-23

**Authors:** Jinho Yoo, Youngbok Lee, Yujung Kim, Sun Young Rha, Yangseok Kim

**Affiliations:** 1 Cancer Metastasis Research Center, Yonsei University College of Medicine, Seoul, Republic of Korea; 2 Brain Korea 21 Project for Medical Science, Yonsei University College of Medicine, Seoul, Republic of Korea; 3 Department of Internal Medicine, Yonsei University College of Medicine, Seoul, Republic of Korea; 4 College of Oriental Medicine, KyungHee University, Seoul, Republic of Korea; 5 Bioinformatics Division, ISTECH Inc., Ilsandong-gu, Goyang-si, Gyeonggi-do, Republic of Korea

## Abstract

**Background:**

Since the completion of the HapMap project, huge numbers of individual genotypes have been generated from many kinds of laboratories. The efforts of finding or interpreting genetic association between disease and SNPs/haplotypes have been on-going widely. So, the necessity of the capability to analyze huge data and diverse interpretation of the results are growing rapidly.

**Results:**

We have developed an advanced tool to perform linkage disequilibrium analysis, and genetic association analysis between disease and SNPs/haplotypes in an integrated web interface. It comprises of four main analysis modules: (i) data import and preprocessing, (ii) haplotype estimation, (iii) LD blocking and (iv) association analysis. Hardy-Weinberg Equilibrium test is implemented for each SNPs in the data preprocessing. Haplotypes are reconstructed from unphased diploid genotype data, and linkage disequilibrium between pairwise SNPs is computed and represented by D', r^2 ^and LOD score. Tagging SNPs are determined by using the square of Pearson's correlation coefficient (r^2^). If genotypes from two different sample groups are available, diverse genetic association analyses are implemented using additive, codominant, dominant and recessive models. Multiple verified algorithms and statistics are implemented in parallel for the reliability of the analysis.

**Conclusion:**

SNPAnalyzer 2.0 performs linkage disequilibrium analysis and genetic association analysis in an integrated web interface using multiple verified algorithms and statistics. Diverse analysis methods, capability of handling huge data and visual comparison of analysis results are very comprehensive and easy-to-use.

## Background

Since the completion of the HapMap project, huge numbers of individual genotypes have been generated from many kinds of laboratories. The efforts of finding or interpreting genetic association between disease and SNPs/haplotypes have been on-going widely, and the necessity of the capability to analyze huge data and diverse interpretation of the result are growing rapidly. Recently developed software programs are well suited for constructing linkage disequilibrium blocks, estimating haplotypes or detecting genetic association between disease and SNPs [1-6]. However, some software programs have drawbacks such as long computation time for the association analysis [1], limited size of dataset [1,2], inconvenient user interface [3-5] and limited number of genetic models or statistics for the association analysis [6]. We have developed an advanced analysis software program, SNPAnalyzer 2.0, which performs sample-specific linkage disequilibrium analysis and implements genetic association analysis using multiple genetic models in an integrated web interface. It can handle hundreds of thousands of SNPs and thousands of samples in a rather manageable time as compared with other software programs.

## Implementation

The analysis engine was developed by C and interface by JAVA, and the operation of the software program is executed using JAVA applet after accessing through a web browser. Although the implementation of the software program is triggered by a web browser, any information about the user's data is not transmitted anywhere because all the analysis are performed locally using JAVA applet. Raw data and all the analyzed results are stored to the user's computer only. If genotypes from two different samples are available, sample-specific analysis and sample-merged analysis are simultaneously implemented in data preprocessing, haplotype estimation and LD blocking. For diverse interpretation of the genetic effects, one allelic or haplotype association test and three genotypic or diplotype association tests are possible. The free implementation of SNPAnalyzer 2.0 and free download of test dataset are available [7].

## Results

SNPAnalyzer 2.0 comprises of four main analysis modules. All the processes are sequentially implemented and results are displayed in comprehensive tables and graphs. The main features and functions are as follows.

### 2.1 Data import

**Figure 1:**
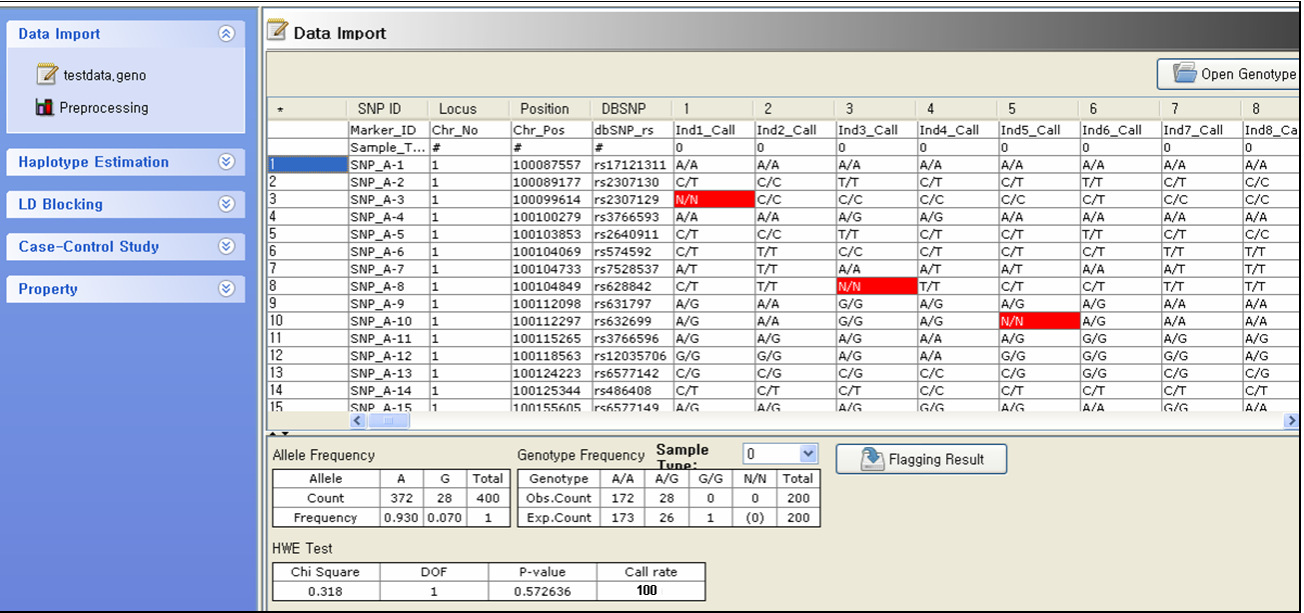
**Individual genotype data import display**. Missing genotypes are represented in red. Allele frequencies, genotype frequencies and p-value of the Hardy-Weinberg Equilibrium test are shown in the table. Triggering tabs for Data Import, Haplotype Estimation, LD Blocking and Case-Control Study are shown on the left panel.

Genotypes of biallelic SNPs should be coded in a tab delimited text file. From the first to the fourth column separately represent marker name, chromosome number, chromosome position and dbSNP rs number of each SNP. Subsequent columns represent individual genotypes of each SNP. First row describes headers and individual identifications. Second row describes sample types, i.e. case sample and control sample that are represented as "0" or "1". Sample type should be in dichotomous number and subsequent rows represent SNPs. Individual genotypes should be coded as allele1, slash and allele2 (e.g. "A/A", "A/G", "G/G"). If input data contains missing genotype, it is coded as "N/N". Detailed information on input data format can be checked in the supplementary information [7]. If data format is correct, data preprocessing is automatically implemented and the results are displayed in the data import interface. Figure 1 shows the result of data importing and data preprocessing.

### 2.2 Data preprocessing

Once the data is input, data quality check and preprocessing is automatically implemented to drop out erroneous SNPs such as monomorphic SNP. SNPs of which minor allele frequencies and missing genotype frequencies are below the specified threshold are also dropped out. Missing genotype can be replaced by heterozygous genotype. Hardy-Weinberg Equilibrium (HWE) test is sequentially implemented to each SNPs, and Bonferroni correction can be applied in the HWE test to prevent excluding SNPs by chance. Red colors in Figure 1 show missing genotypes. Allele frequencies, genotype frequencies, and the result of the HWE test are displayed in tables.

### 2.3 Haplotype estimation

**Figure 2:**
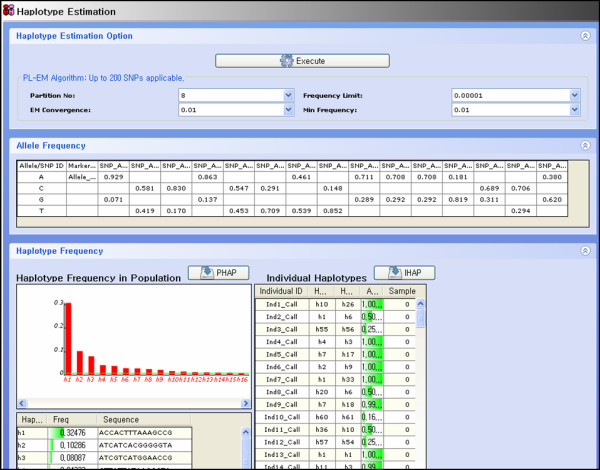
**Haplotype estimation display**. Haplotype estimation can be implemented in parallel using two different algorithms like EM-based algorithm and PL-EM algorithm. The upper panel shows the control options for PL-EM algorithm. Middle panel shows the observed alleles and allele frequencies. The histogram and tables in the bottom panel shows the most likely haplotypes and their frequencies in a given sample. Individual haplotypes and estimation accuracies are shown on the right part of the bottom panel.

A haplotype is a particular pattern of alleles at sequential loci on a single chromosome. In order to reconstruct haplotypes from the unphased diploid genotype data, we have used EM-based algorithm [8] and PL-EM algorithm [9]. For the performance of reconstruction, 25 or less SNPs are recommended for the EM-based algorithm. PL-EM algorithm can analyze more than 25 SNPs. Reconstructed haplotypes are displayed in an integrated interface (Figure 2). The most likely haplotypes and their frequencies in a given sample are displayed in histogram and table. Reconstructed individual haplotypes and accuracies of the reconstruction are displayed in a separate table. The sample-specific analysis result can be saved as a tab delimited text file.

### 2.4 Linkage disequilibrium (LD) blocking

**Figure 3:**
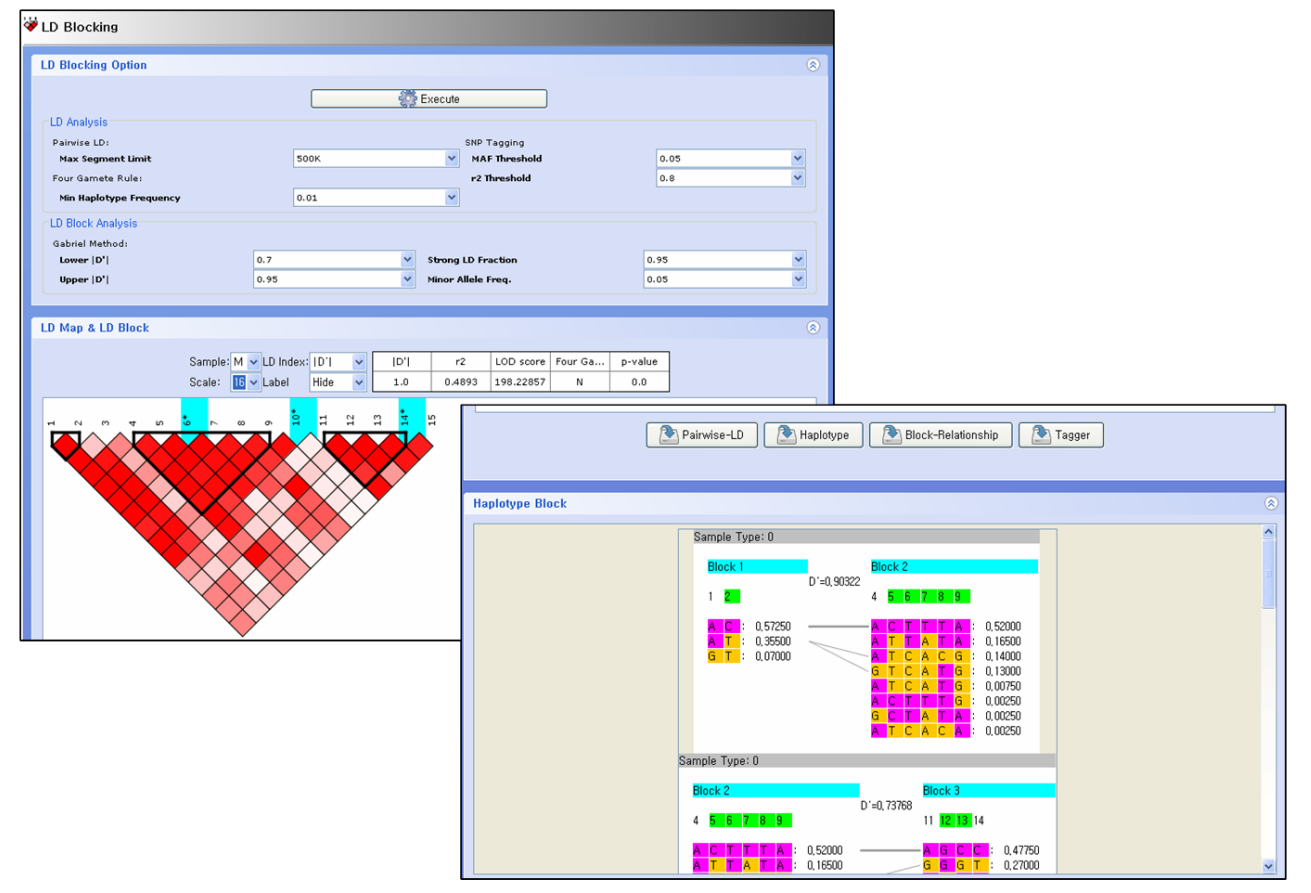
**LD blocking display**. The upper panel shows the control options for LD blocking. The following panel shows the linkage disequilibrium pattern. The part in deep red means that there exists strong pairwise linkage disequilibrium between adjacent SNPs and the area enclosed by a thick black line designates LD block. Tagging SNPs are represented as light blue bars up in the linkage disequilibrium pattern graph. Haplotypes, haplotype frequencies and multi-allelic D' estimated in the adjacent LD blocks are displayed in the bottom of the linkage disequilibrium pattern graph.

**Figure 4:**
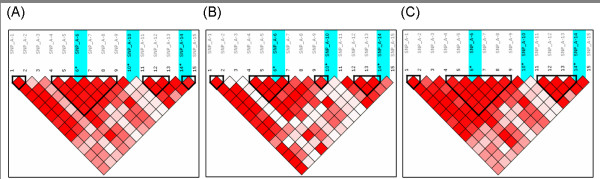
**Different patterns of linkage disequilibrium of two samples**. (A) Control sample, (B) Case sample, (C) Merged sample.

The degree of genetic linkage between two different SNPs can be estimated by several linkage disequilibrium indices like D', r^2^, LOD score, or by four gamete test [10]. Representative SNP that has strong correlation (r^2 ^> 0.8) with other SNPs is designated as pairwise tagging SNP. The entire pattern of linkage disequilibrium and tagging SNPs are displayed in a reverse triangle. Several SNPs that are in strong linkage disequilibrium can be bound into one LD block and we construct LD blocks using Gabriel's method [11]. Crossover percentages between haplotypes that are reconstructed within adjacent LD blocks and multi-allelic D' are simultaneously calculated. Figure 3 shows the linkage disequilibrium pattern, LD blocks and reconstructed haplotypes. Red color in the linkage disequilibrium pattern graph means that there exists strong pairwise linkage disequilibrium between adjacent SNPs, and the area enclosed by a thick black line shows LD block. Haplotypes, haplotype frequencies and multi-allelic D' are displayed in the bottom of the linkage disequilibrium pattern graph. Figure 4 shows the different linkage disequilibrium patterns of two samples and merged sample.

### 2.5 Association analysis

**Figure 5:**
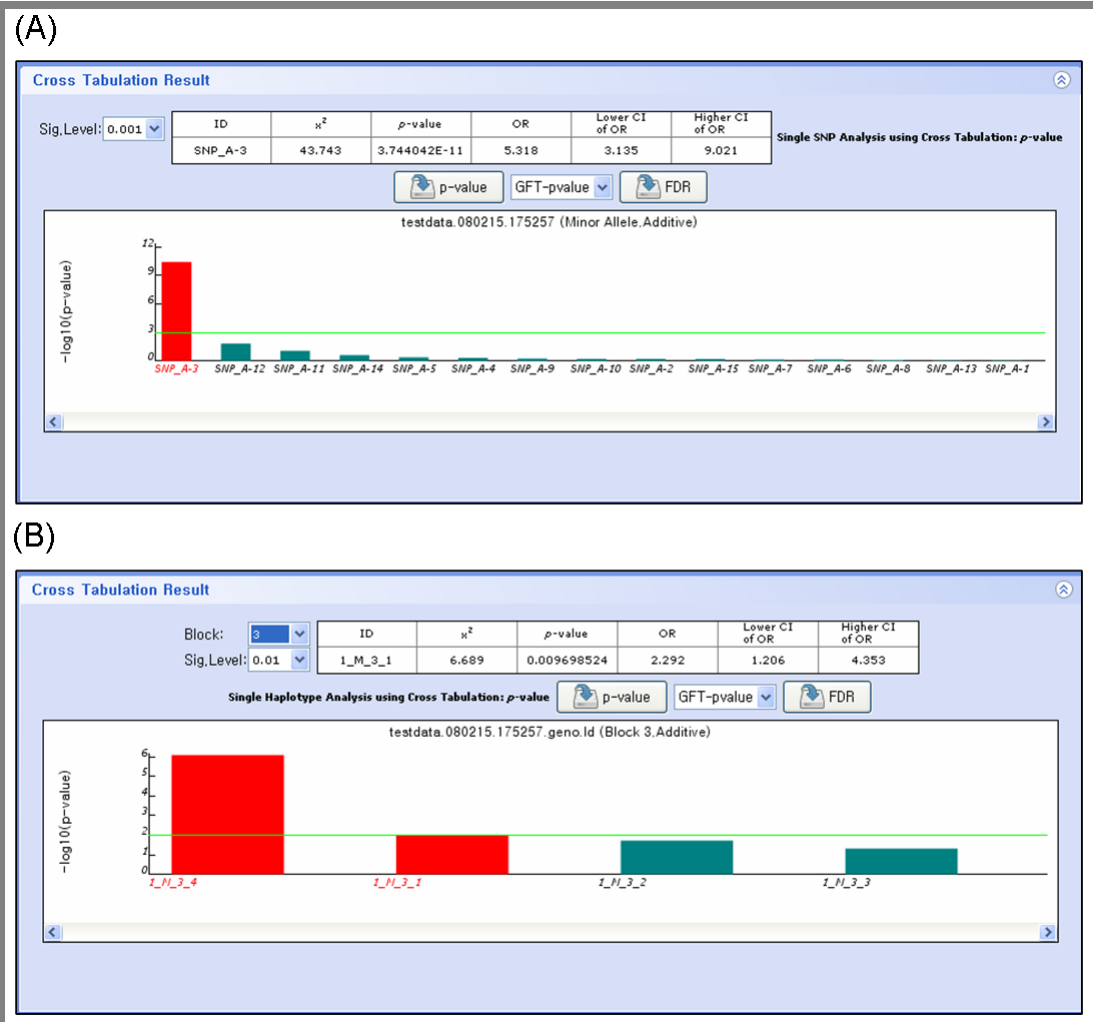
**Case-control study display**. The p-values from association analysis (A) with 15 SNPs and (B) with four haplotypes in the third LD block are separately shown in the bar charts. P-values are log transformed and sorted by descending order. Odds ratios (OR) and 95% confidence interval of odds ratios are displayed simultaneously with p-value of the chi-square test in the upper table. Green horizontal line represents significance level.

Genetic association between disease and SNP is analyzed using Pearson's chi-square test if the input data contains two different samples such as case sample and control sample. We applied goodness of fit test and likelihood ratio test simultaneously to avoid biased results acquired by applying only a single statistics. False positive control is implemented by both Bonferroni correction and false discovery rate [12]. Odds ratios (OR) and 95% confidence interval of odds ratios are calculated simultaneously with chi-square test. If there are haplotypes reconstructed from haplotype estimation or LD blocking, genetic association analysis between disease and haplotypes is performed using the same statistics as SNPs. For analyzing different genetic effects conveniently, four genetic models are available. Additive model deals with allelic or haplotype association, and genotypic or diplotype association can be analyzed using codominant model, dominant model or recessive model. Figure 5 shows the result of association analysis with SNPs and haplotypes. Bar chart displays the log transformed p-values that are sorted by descending order.

In the association analysis with haplotypes, we applied a haplotype-specific test with one degree-of-freedom. Estimation of haplotype effects was not implemented because the current version handles only the haplotype frequencies previously reconstructed in the LD blocking analysis. Several algorithms for estimating haplotype effects have been developed by many researchers [13-16]. Software programs like THESIAS [17] and Haplo Stats [18] are freely available and widely used for the analysis of haplotype effects.

### 2.6 Data export

**Figure 6:**
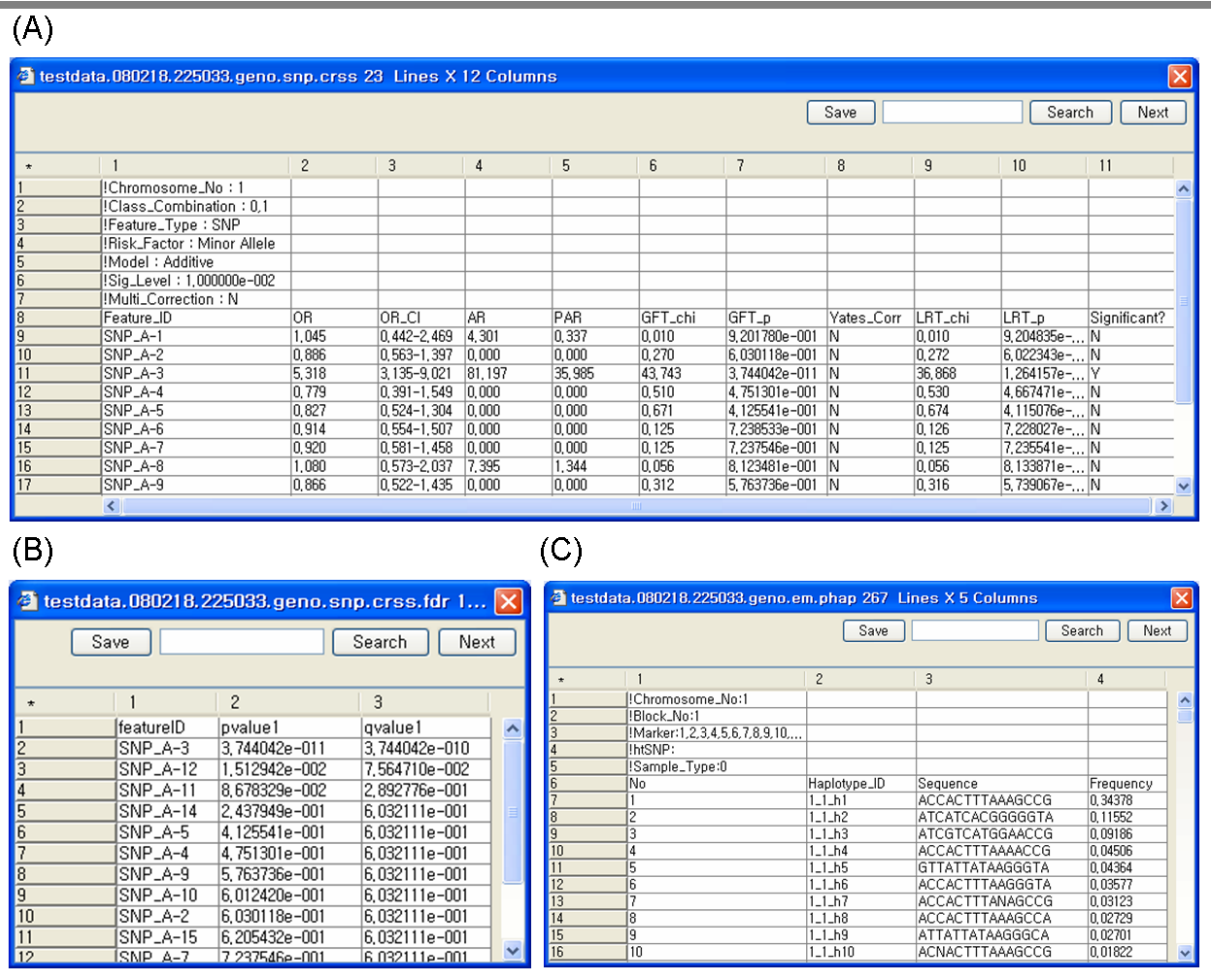
**Data export display**. All the analyzed results can be saved as tab delimited text files for user's convenience: (A) association analysis, (B) false discovery rate, (C) reconstructed haplotypes.

All the analyzed results can be saved as tab delimited text files for user's convenience. Figure 6 shows the results of association analysis, false discovery rate and reconstructed haplotypes.

### 2.7 Accuracy measure

For the measurement of the accuracy of the haplotype estimation, we applied two methods [2,19]: (i) the accuracy measured with the average error rate and (ii) the discrepancy between the true haplotype frequencies and the estimated haplotype frequencies. The average error rate is the ratio of the number of incorrectly reconstructed samples to the total number of samples. The discrepancy was calculated using index *D*, given as $D=\sum_{j}|{\hat{f}}_{j}-f_{j}|/2$, where ${\hat{f}}_{j}$ is the estimated haplotype frequency and *f*_*j *_is the true haplotype frequency of the *j*th sample. The true haplotype datasets were obtained from the dbSNP database at NCBI [20], and the detailed description of the data is available in the supplementary material [See Additional file 1]. The number of samples and SNPs are summarized in Table 1 and all the test datasets are downloadable [7]. For the African American group, 14 haplotypes were correctly estimated of the 15 true haplotypes by EM-based algorithm. PL-EM algorithm estimated 15 true haplotypes perfectly. For the Asian American group, 13 haplotypes were correctly estimated of the 15 true haplotypes by EM-based algorithm and by PL-EM algorithm. All the mismatched haplotypes were rare haplotypes with population frequencies less than 1.5%. For both African American group and Asian American group, the haplotypes of only two individuals were incorrectly reconstructed by both algorithms. Table 2 shows the accuracies of the haplotype estimation for the EM-based algorithm and PL-EM algorithm employed by SNPAnalyzer 2.0.

**Table 1:** The numbers of individuals of each ethnic group and the numbers of SNPs used for redefining haplotypes

Ethnic group	Afr	Asi
Sample no.	72	75
SNP no.	22	22

African American and Asian American ethnic groups are denoted as Afr and Asi, respectively.

**Table 2:** The accuracies of haplotype estimation produced by SNPAnalyzer 2.0

Ethnic group	Afr		Asi	
		
Error type	DIS	AER	DIS	AER
EM	0.020	0.027	0.020	0.027
PL-EM	0.000	0.000	0.022	0.027

African American and Asian American ethnic groups are denoted as Afr and Asi, respectively. EM and PL-EM represent EM-based algorithm and PL-EM algorithm, respectively. AER represents average error rate, and DIS represents discrepancy.

**Figure 7:**
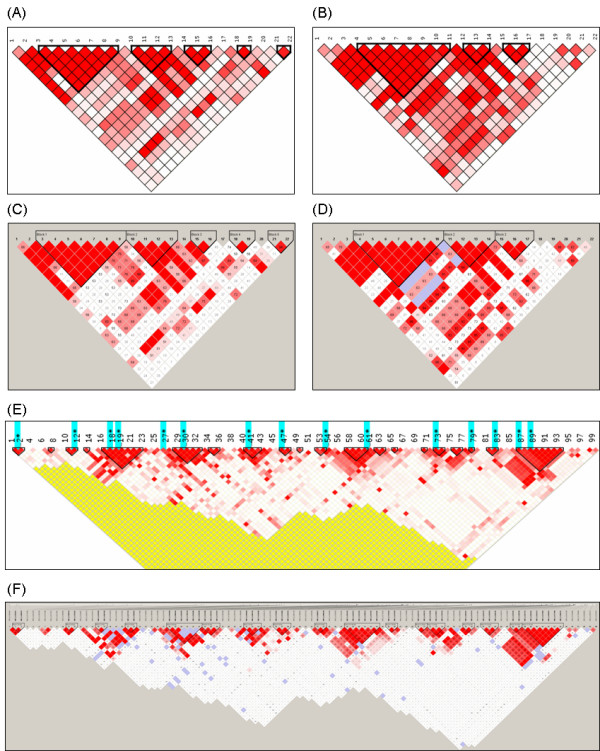
**Comparison of LD blocking**. The results of LD blocking of (A) African American ethnic group and (B) Asian American ethnic group are produced by SNPAnalyzer 2.0, and (C) African American ethnic group and (D) Asian American ethnic group by Haploview program. The structures of LD blocks consisting of 100 SNPs are produced by (E) SNPAnalyzer 2.0 and by (F) Haploview program.

For the reliability of LD blocking, we compared the results produced by SNPAnalyzer 2.0 with the results by Haploview program [6]. Figure 7 shows the results of LD blocking. For the African American group, all the LD blocks produced by two software programs were the same, except the third block. For the Asian American group, the structure of the second LD block was mismatched. The overall structures of the LD blocks are similar by both programs when using the simulated genotype dataset consisting of 100 SNPs and 135 samples. All the test data are downloadable [7].

### 2.8 Performance

The performance of the software program was tested by computation time in seconds according to the numbers of SNPs and samples used for association analysis. For the performance test, we simulated several genotype datasets having different number of SNPs. All the simulated datasets contained 1,000 control samples and 1,000 case samples. Two other publicly available software programs, BEAGLE [3] and PLINK [4], were used for comparison. Table 3 shows the results of computation time. The computation time increased linearly with the increasing number of SNPs. SNPAnalyzer 2.0 was slightly faster than two other software programs in spite of the fact that it created graphic results as well as statistical results. PLINK and BEAGLE programs created text files only containing statistical results.

**Table 3:** The computation time for association analysis

Software program	Number of SNPs					
	
	1000	5000	10000	20000	50000	100000
SNPAnalyzer 2.0	2	10	21	42	104	208
BEAGLE	3	12	24	47	118	235
PLINK	5	23	47	96	237	472

The time unit is seconds and we used a desktop with a 3.0 GHz processor and 2 GB memory as a test bed.

The limit of the analyzable dataset size depends on the random access memory (RAM) of user's computer. We checked that the association analysis using genotype data with over 100,000 SNPs and 2,000 samples was possible. All the test datasets are downloadable [7].

## Discussion

In the past work, we have developed a software program that calculates linkage disequilibrium between SNPs, reconstructs haplotypes and performs quantitative trait analysis [2]. To meet the increasing demand for whole-genome association study, we have developed SNPAnalyzer 2.0 that can handle the genetic linkage disequilibrium analysis and the genetic association analysis between disease and SNPs/haplotypes in an integrated web interface. For the accuracy of the analysis, it implements several verified algorithms and statistics. The accuracy of the haplotype estimation was very high and the results of LD blocking were similar both by SNPAnalyzer 2.0 and Haploview program [6]. Some mismatched structures of LD blocks are due to the different usage of the detailed parameters or algorithms applied by each software programs. For example, Haploview program used an accelerated EM algorithm. However, SNPAnalyzer 2.0 used both the EM-based algorithm and PL-EM algorithm for haplotype estimation. Comparison among control, case and merged samples is possible for linkage disequilibrium analysis using many LD indices. False positive control is implemented by multiple test correction and false discovery rate (FDR) in the association analysis. All the results are provided as tab delimited text files for user's convenience. We plan to implement more statistical analysis in future versions: stratification analysis, interaction analysis using multiple SNPs, haplotype effects analysis, and classification analysis for multiple samples.

## Conclusion

SNPAnalyzer 2.0 performs linkage disequilibrium analysis and genetic association analysis in an integrated web interface. It implements multiple verified algorithms and statistics for the enhanced reliability of the analysis. Visual comparison and interpretation of the analysis result between two different sample groups are very comprehensive. The allelic or haplotype association and genotypic or diplotype association can be analyzed using multiple genetic models. Hundreds of thousands of SNPs and thousands of samples are analyzable in moderate time, and the analysis results are displayed in figures and tables for user's convenience.

## Availability and requirements

Project name: SNPAnalyzer 2.0

Project homepage: 

Operating systems: Windows

Programming language: C and JAVA

Web application: Internet Explorer 6.0 or higher (Internet connection required for program installation)

License: free non-commercial research use license

Any restrictions to use by non-academics: none

## Authors' contributions

JY contributed to the design of the analysis engine and interface, and drafted the manuscript. YL and YK coded and implemented the whole part of the SNPAnalyzer 2.0. SYR provided helpful comments on the development of the software and test. YK supervised the project. SYR and YK were involved in revising the manuscript. All authors read and approved the final manuscript.

## Supplementary Material

Additional file 1 This file contains the description of the true haplotype data obtained from the dbSNP database at NCBI . Click here for file
