# Supplementary material for: SNPAnalyzer 2.0: A web-based integrated workbench for linkage disequilibrium analysis and association analysis
Source: BMC Bioinformatics. 2008 Jun 23;9:290. doi: 10.1186/1471-2105-9-290 (PMC2453143; doi:10.1186/1471-2105-9-290)
Supplement: Additional file 1 — This file contains the description of the true haplotype data obtained from the dbSNP database at NCBI . [file 1471-2105-9-290-S1.doc]

**1. True haplotype data**

We obtained haplotype data from the dbSNP database at NCBI (http://www.ncbi.nlm.nih.gov/SNP/). Haplotypes are for the HLA gene region where traditional serological alleles have been defined at the molecular (SNP) level. Haplotypes from African American and Asian American ethnic groups were selected for analysis from five possible ethnic groups, being African American, Asian American, Caucasian, Latin American and Native American. The African American group comprises 72 individuals and the Asian American group comprises 75 individuals. Family histories were not considered in our analysis. Each group’s haplotypes were redefined by using only biallelic SNPs, and individual’s genotypes were reconstructed from these redefined haplotypes.
